# Addition of ultrasound to mammography in the case of dense breast tissue: systematic review and meta-analysis

**DOI:** 10.1038/s41416-018-0080-3

**Published:** 2018-05-08

**Authors:** Matejka Rebolj, Valentina Assi, Adam Brentnall, Dharmishta Parmar, Stephen W. Duffy

**Affiliations:** 1 Centre for Cancer Prevention, Wolfson Institute of Preventive Medicine, Barts & The London School of Medicine and Dentistry, Queen Mary University of London, Charterhouse Square, London, EC1M 6BQ UK; 2 Edinburgh Clinical Trials Unit, Usher Institute of Population Health Sciences and Informatics, University of Edinburgh, Edinburgh, UK

**Keywords:** Breast cancer, Health care

## Abstract

**Background:**

Mammography is less effective in detecting cancer in dense than in fatty breasts.

**Methods:**

We undertook a systematic search in PubMed to identify studies on women with dense breasts who underwent screening with mammography supplemented with ultrasound. A meta-analysis was undertaken on the proportion of cancers detected only by ultrasound, out of all screen-detected cancers, and the proportion of women with negative mammography who were referred for assessment following ultrasound screening.

**Results:**

Twenty-nine studies satisfied our inclusion criteria. The proportion of total cancers detected only by ultrasound was 0.29 (95% CI: 0.27–0.31), consistent with an approximately 40% increase in the detection of cancers compared to mammography. In the studied populations, this translated into an additional 3.8 (95% CI: 3.4–4.2) screen-detected cases per 1000 mammography-negative women. About 13% (32/248) of cancers were in situ from 17 studies with information on this subgroup. Ultrasound approximately doubled the referral for assessment in three studies with these data.

**Conclusions:**

Studies have consistently shown an increased detection of breast cancer by supplementary ultrasound screening. An inclusion of supplementary ultrasound into routine screening will need to consider the availability of ultrasound and diagnostic assessment capacities.

## Introduction

Since the publication of the randomised trials showing a significant breast cancer mortality reduction with the offer of breast screening with mammography, large numbers of screening programmes have been instituted worldwide.^1–4^ These programmes are estimated to prevent substantial numbers of breast cancer deaths^2–4^ and standards have been developed to monitor and maintain the quality of the services.^5,6^

One area where there is room for improvement is the lower sensitivity of mammographic screening in women with dense breast tissue.^7^ Since the introduction of legislation in the USA requiring disclosure of mammographic density to screenees, there has been considerable international interest in potential variation in screening regimen based on breast density.^8,9^ Possible tactics include increased frequency of screening in the case of dense breast tissue,^10^ but in both the USA and Europe, there is much interest in supplemental imaging in addition to mammography.^8,11,12^ The latter option seems logical, since if a test is shown to be less sensitive in a population, using a different test may be more effective than applying the same test more frequently.

While there is strong evidence that magnetic resonance imaging confers a substantial improvement in sensitivity, particularly in high-risk groups,^13^ it remains an expensive option and requires considerable commitment from the screenee.^14^ There is therefore interest in the use of ultrasound, hand-held or automatic, in addition to mammography in the case of dense breast tissue.^12,15^ A policy decision regarding the use of adjunctive ultrasound for screening in dense breasts would need to be informed by evidence on the increase in breast cancer detection capability, the resource and human costs of the ultrasound imaging, and the resource and human costs of further diagnostic workup as a result of positive ultrasound findings. A decision would also need to be made as to how to define the dense tissue subgroup of the population, as there are many methods of measuring breast density.^7^

In this paper, we review the published literature on the use of ultrasound in addition to mammography in screening women with dense breast tissue. We summarise in quantitative terms the likely benefit in terms of increased breast cancer detection, and the effect on the increased diagnostic activity, specifically in terms of recall rates for assessment. The benefit and the required diagnostic activity are further discussed in the context of a routine mammography screening service such as the one implemented by the NHS Breast Screening Programme.

## Materials and methods

### Inclusion criteria and PICOS terms

Methods and inclusion criteria were specified in advance, although the protocol was not registered. Studies had to report data on breast cancers, either invasive or ductal carcinomas in situ (DCIS), detected in consecutive or randomly selected women with dense breasts. No limitation was imposed for the women’s age, the breast density classification system used in the study, or the proportion of the included women who had additional breast cancer risk factors. These women were screened with mammography and had undergone supplemental screening with ultrasound, the latter at least in case mammography was negative.

As the focus was on the detection at screening, we excluded studies of women with symptoms, and any cancers diagnosed after normal screening tests (i.e., interval cancers). We also excluded studies where women receiving mammography screening were different from women receiving ultrasound examinations, or where breast cancers in dense breasts were not reported separately from those in fatty breasts. Studies published before year 2000 were excluded as the ultrasound imaging technology has developed considerably in terms of quality in recent decades.

No language restrictions were imposed. In case of duplicate publications, the report with the most complete data was included in the meta-analysis.

### Literature search

The search was developed by D.P. and S.D. The investigators searched PubMed on 29 June 2016 using the following criteria: [ultrasound AND breast AND screening AND (“density” OR “dense”)], limited to publication date from 1 January 2000 onward. The search was updated on 26 July 2017 to identify any new publications since 1 June 2016. All analyses were based on published data, but study authors were contacted, if necessary, for further clarifications that concerned study eligibility.

Two authors (S.D., M.R.) independently screened the abstracts of all retrieved records, with a subgroup also screened by D.P. Reference lists of all reviews and other types of secondary publications (including letters, news items, etc.) were checked for additional primary data. Two authors (either S.D. or V.A., and M.R.) independently assessed full texts for inclusion and retrieved information on study and population characteristics and on screening outcomes into pre-specified tables. Two authors (V.A., M.R.) independently evaluated study quality following the Quality Assessment of Diagnostic Accuracy Studies (QUADAS) Version 2 evaluation tool.^16^ Any discrepancies were resolved through consensus.

### Statistical analysis

The primary aim was to measure the relative increase in cancer detection from supplemental ultrasound screening. For this purpose, we considered those studies reporting the number of cancers detected only by ultrasound (*r*) and the total number of cancers detected (*n*, by mammography and ultrasound supplemental screening). A meta-analysis was undertaken on the proportion *p* = *r*/*n* detected only by ultrasound. This may be related to the percentage increase through *q* = *r*/(*n*−*r*) = (*p*^−1^−1)^−1^. To help stabilise the binomial variance, an arcsin (*r*/*n*)^0.5^ transformation was applied,^17^ on which scale standard fixed-effect (FE) (inverse variance) and random-effect (RE) meta-analysis estimates were obtained.^18^ Results were back-transformed to proportions for presentation; exact confidence intervals for individual studies were presented in forest plots. Evidence for departure from the FEs model was assessed using the *I*^2^ statistic (ratio of between-study variance to total variance). Funnel plot is a standard visual instrument examining the relationship between the effect estimate and a measure of study precision in order to investigate potential reporting or other biases.^19^ As study sizes and standard errors were not reported for all studies, we examined *p* against total number of cancers on a square root scale, centred around an overall *p* from the total number of cancer detected only by ultrasound divided by total cancers. Prediction intervals were obtained from the inverse binomial transformation, and plotted using a loess smoother to aid interpretation.

The secondary aim was to determine the additional detection of breast cancer and referrals for assessment per 1000 women with mammography negative results. A meta-analysis was undertaken on the absolute numbers of detected cancers and referrals owing only to ultrasound examinations. The 95% confidence intervals were calculated as exact binomial intervals.

Furthermore, we investigated the associations between the variables of interest using Pearson correlation coefficients (*ρ*) weighted by study size (number of all screened women or number of women with negative mammography).

In the studies where the same women underwent several screening rounds, the unit of observation was an individual screening episode. There were no pre-defined sub-groups.

Analysis was undertaken using the meta and weights packages for statistical software R 3.4.1.^20–22^

## Results

### Search results

**Fig. 1:**
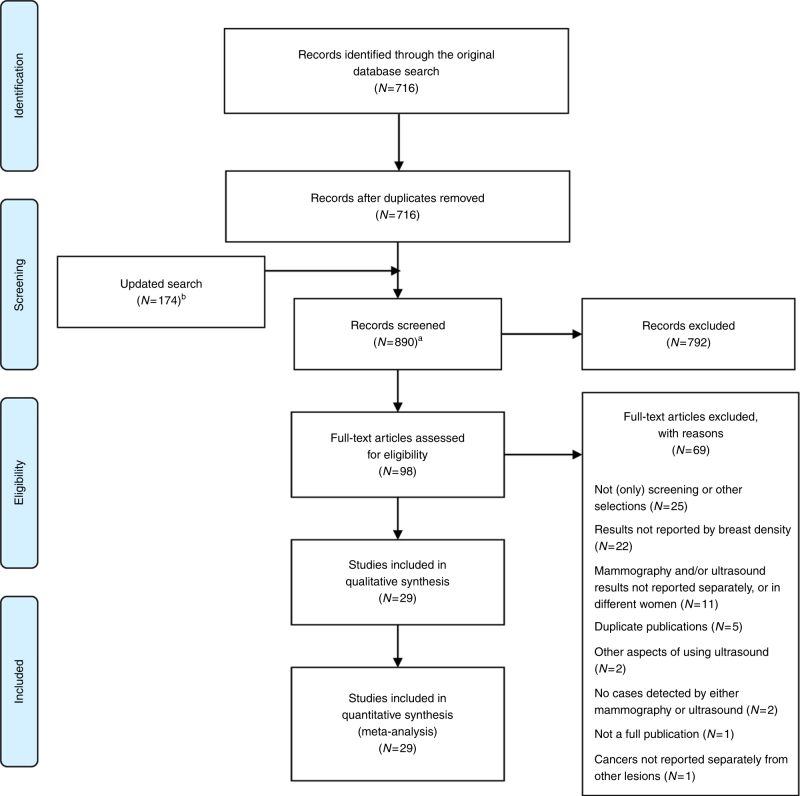
PRISMA flow diagram of study selection. Baseline search undertaken on 29 June 2016. Update search undertaken on 26 July 2017. ^a^ Reference lists of reviews and similar publications were examined for any additional studies reporting primary data. The latter studies were included in the counts of articles assessed for eligibility, and, if they satisfied the inclusion criteria, they were included in the meta-analysis. ^b^ This number may have included duplicate records compared to the original search. No new studies reporting primary data were identified through reviews and similar secondary publications in the updated search, suggesting that the pool of the relevant studies had been exhausted

The original search identified 716 unique records (Fig. 1). The updated search identified 174 records. In total, 29 studies satisfied our inclusion criteria. Although several reviews had been published,^23,24^ no previous meta-analysis could be identified.

In total, 13 studies compared mammography and supplemental ultrasound screening to screening using mammography alone in the same women.^12,15,25–35^ Ten of these studies were undertaken in general populations of women with dense breasts^15,25–31,34,35^ and three studies were in women with additional risk factors^12,32,33^ (Table 1). An additional 16 studies were undertaken using ultrasound in women with negative mammography,^8,36–50^ of which one^46^ was in women with additional risk factors. Ten studies were undertaken in the USA,^8,15,27,40,44–47,49,50^ six in Italy,^25,30,31,33,36,39^ five in South Korea,^34,37,41,43,48^ and one each in China,^26^ Israel,^38^ Singapore,^42^ Austria,^28^ Thailand,^29^ Germany^32^ and Sweden,^35^ and one study was undertaken in multiple countries (USA, Argentina, Canada).^12^ All studies with reported data on age also included women below 50 years, but age breakdowns for the studied outcomes were not reported systematically. Twelve studies reported women undergoing a clinical breast examination prior to an ultrasound examination^8,27,30,32–34,38–42,46^ (Supplementary Table 1). As reported, mammography was read with knowledge of ultrasound imaging in three studies^29,37,39^ but in another three studies the interpretation of ultrasound imaging was blinded to mammographic findings.^12,32,33^ Screening was undertaken either in organised programmes or in other settings, e.g. allowing women and/or their doctors to self-refer.

**Table 1:** General description of the studies included in the meta-analysis

**Authors, publication year**	**Screening population**	**Additional risk factors** ^**a**^	**Description of the studied population**	**Exclusion criteria**	**Definition of dense breasts**	**Type of MX**	**Type of US**
Buchberger et al^28^	All	No	Undergoing screening	Cyst, recent MX + or PE + , MX + by a second reader	BI–RADS 2–4	Screen-film	Hand-held
Kuhl et al^32^	All	Yes	Asymptomatic women	NR	BI–RADS 3–4	Screen-film	Hand-held
Kaplan^40^	MX-	No	Asymptomatic women presenting for screening MX	NR	BI–RADS 3–4	Film-screen	Hand-held
Kolb et al^27^	All	No	Asymptomatic women	Symptoms on prior CBE	BI–RADS 2–4	Screen-film	Hand-held
Crystal et al^38^	MX-	No	Asymptomatic women	Cancers whose retrospectively reviewed MX revealed a visible mass or were determined to be palpable on re-examination by a surgeon	BI–RADS 2–4	Film-screen	Hand-held
Brancato et al^30^	All	No	Asymptomatic women self-referring to MX outside of the population-based screening programme	US performed in >1 month	BI–RADS 3–4	NR	Hand-held
De Felice et al.,^31^	All	No	Routine MX examination, spontaneously requested	NR	BI–RADS 3–4	Screen-film	Hand-held
Sardanelli et al.,^33^	All	Yes	Asymptomatic women	<25 years, pregnancy, lactation, current chemotherapy, terminal illness, contraindication to MR imaging	>50% fibroglandular density	Screen-film	Hand-held
Weinstein et al^46^	MX-	Yes	Research screening	NR	BI–RADS 3–4	Film-screen	Hand-held
Bae et al^34^	All	No	Asymptomatic women with non-palpable breast cancer	MX findings at review identified as a correlate of US-detected breast cancer, no treatment	BI–RADS 3–4	NR	Hand-held
Corsetti et al^25^	All	No	Self-referring to screening	Symptoms	BI–RADS 3–4	Screen-film	Hand-held
Youk et al., ^41^	MX-	No	Asymptomatic women undergoing general screening	No surgical biopsy, not confirmed by a surgical biopsy, did not have at least a 2-year follow-up US	BI–RADS 3–4	Screen-film	Hand-held
Berg et al., ^12^	All	Yes	Asymptomatic women presenting for routine MX	Pregnancy or lactation, metastatic disease, symptoms, surgery in ≤12 months, implants	BI–RADS 3–4 in ≥1 quadrant	Both (either-or)	Hand-held
Hooley et al., ^8^	MX-	No	Screening US breast examinations following the legal change in breast density notification	Bilateral mastectomy, most recent MX >12 month or none	BI–RADS 3–4	Digital	Hand-held
Leong et al., ^42^	MX-	No	Asymptomatic women undergoing routine MX	NR	BI–RADS 3–4	Digital	Hand-held
Weigert and Steenbergen, ^44^	MX-	No	Screening US breast examinations following the legal change in breast density notification	NR	BI–RADS 3–4	NR	Hand-held
Girardi et al., ^39^	MX-	No	Asymptomatic self-referring women	Symptoms, examination at other institutions	BI–RADS 3–4	Digital	Hand-held
Wang et al., ^26^	All	No	Rural women with screen-detected cancer who accepted MX and US before treatment	Missing MX	BI–RADS 3–4	NR	Hand-held
Korpraphong et al., ^29^	All	No	Asymptomatic women undergoing voluntary screening	Symptoms, history of breast cancer, previous atypical ductal hyperplasia, atypical lobular hyperplasia or LCIS	BI–RADS 2–4	Digital	Hand-held
Brem et al., ^15^	All	No	Asymptomatic women attending for MX screening	Symptoms, procedures or treatment in ≤1 year, pregnancy or lactation, discordant breast density classification technician vs. radiologist	BI–RADS 3–4	Digital	Automated
Chang et al^37^	MX-	No	Asymptomatic women seeking prevalence breast screening	No follow-up >12 after screening, history of breast or ovarian cancers, chest irradiation, BRCA positive, positive or suspicious MX	BI–RADS 3–4	Digital	Hand-held
Hwang et al^43^	MX-	No	Asymptomatic women undergoing screening	Symptoms, no follow-up ≥1 year after screening	BI–RADS 3–4	Digital	Hand-held
Weigert and Steenbergen^45^	MX-	No	Screening US breast examinations following the legal change in breast density notification	NR	BI–RADS 3–4	NR	Hand-held
Kim et al^48^	MX-	No	Consecutive women undergoing screening with MX and US	>1 US in 1 year with normal prior US, known risk factors other than dense breasts, no surgery or follow-up in 12 months	BI–RADS 3–4	Digital	Hand-held
Tagliafico et al^36^	MX-	No	Asymptomatic women self-referring for MX screening	History of breast cancer, pregnancy, lactation, implants	BI–RADS 3–4	Digital	Hand-held
Wilczek et al^35^	All	No	Asymptomatic women invited for service screening MX	Currently pregnant, breastfeeding, previous breast surgery, history of breast cancer diagnosis and/or treatment in past 12 months	BI–RADS 3–4	Digital	Automated
Destounis et al^50^	MX-	No	Screening US breast examinations following the legal change in breast density notification	Symptoms	BI–RADS 3–4	Digital	Hand-held
Klevos et al^49^	MX-	No	Asymptomatic women undergoing routine MX	≥20% lifetime risk of breast cancer, personal history of breast cancer	BI–RADS 3–4	Digital	Hand-held
Weigert^47^	MX-	No	Screening US breast examinations following the legal change in breast density notification^b^	NR	BI–RADS 3–4	NR	Hand-held

*BI-RADS 2* density: breasts with scattered areas of fibroglandular density (sometimes defined as 25–50% fibroglandular tissue), *BI-RADS 3* density: breasts with heterogeneously dense tissue (50–75%), *BI-RADS 4* density: breasts with extremely dense breast tissue (>75%), *MX* mammography, *NR* not reported, *US* ultrasound

^a^Women with risk factors other than dense breasts may have been included in all studies, in variable proportions. A study was categorised as “Yes” if the additional risk factors were a selection criterion for inclusion.

^b^The first two years of screening with ultrasound were excluded from this review, as the data were already reported in the two previous publications.^44,45^

Breast density was defined predominantly using the American College of Radiology’s Breast Imaging–Reporting and Data System (BI–RADS). Four studies defined dense breasts as BI–RADS categories 2 to 4 (i.e., including breasts with ≥25% fibroglandular tissue),^27–29,38^ whereas 24 studies defined dense breasts as BI–RADS 3 or 4 (i.e. including breasts with ≥50% fibroglandular tissue).^8,12,15,25,26,30–32,34–37,39–50^ One study reported classifying breasts as dense if fibroglandular tissue occupied >50% of the breast as a mean of two mammographic views but did not explicitly explain the classification system.^33^

### Extra detection of breast cancers

**Fig. 2:**
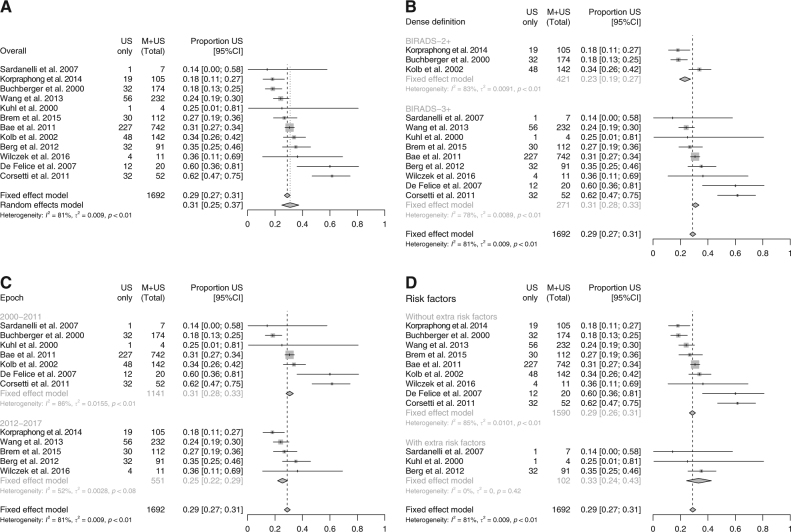
Additional detection of breast cancer cases with ultrasound in mammography negative women, compared to the detection with stand-alone mammography (based on 12 studies reporting detection by both screening modalities). BI-RADS 2 density: breasts with scattered areas of fibroglandular density (sometimes defined as 25–50% fibroglandular tissue). BI-RADS 3 density: breasts with heterogeneously dense tissue (50–75%). BI-RADS 4 density: breasts with extremely dense breast tissue (>75%). CI confidence interval, M mammography, US ultrasound (**a**) Additional detection, overall results. (**b**) Additional detection, by definition of breast density. (**c**) Additional detection, by year of study. (**d**) Additional detection, by whether the study focused on women with additional risk factors

The main analysis included 1692 breast cancers detected in 12 studies reporting detection of breast cancer in the entire screening population, of which 494 (29%) were detected only by supplemental ultrasound (a relative detection rate of 141%, with the increased detection calculated as 494/(1692–494), see Statistical Analysis and Table 2). The overall FE estimate of the proportion of total cancers detected by ultrasound was 0.29 (95% CI: 0.27–0.31); the estimate of an RE distribution mean was 0.31 (95% CI: 0.25–0.37). Both measures were very close despite substantial between-study variation (*I*^2^ = 81% (95% CI: 68–89%); Fig. 2a). The results suggest that detection rates are on average increased by approximately 40% with supplemental ultrasound compared to mammography alone. In the only six studies reporting detection separately for DCIS and invasive cases, DCIS cases represented only a smaller proportion of the cases detected by ultrasound. The FE estimate, almost identical to the RE estimate, was 0.10 (95% CI: 0.05–0.16), consistent with an increase in the detection of 11%. The study by Brancato and colleagues^30^ was not included as the total number of cancers in women undergoing supplemental ultrasound screening was unknown; a sensitivity analysis where it was included did not materially alter the results.

**Table 2:** Study outcomes

**Study**	**Mammography** ^**f**^			**Ultrasound in MX- women** ^**f**^			**Positive screening outcomes**				
	**N screens**	**Detected cancers**		**N screens**	**Additionally detected cancers**		**Type (threshold)** ^**e**^	**Mammography**		**Ultrasound in MX- women**	
		**N (DCIS)**	**Per 1000**		**N (DCIS)**	**Per 1000**		**N**	**Per 1000**	**N**	**Per 1000**
**Whole screening population**											
Buchberger et al^28^	8970	142 (47)	15.8 (5.2)	8103	32 (5)^g^	3.9 (0.6)	NR	NR	NR	NR	NR
Kuhl et al^32^	NR	3 (1)	NR	NR	1 (1)	NR	NR	NR	NR	NR	NR
Kolb et al^27^	13,547	94	6.9	12,193	48	3.9	Biopsy (actual)	423	31	320	26
Brancato et al^30^	26,973	156^a^	5.8	5227	2	0.4	Test + (U3–5)	NR	NR	108	21
							Test + (U4–5)	NR	NR	23	4
							Biopsy (actual)	NR	NR	29	6
De Felice et al^31^	NR	8	NR	1754	12	6.8	Test + (U3–5)/Biopsy (rec)	NR	NR	187	36
Sardanelli et al^33^	NR	6 (1)	NR	NR	1 (0)	NR	NR	NR	NR	NR	NR
Bae et al^34^	NR	515	NR	NR	227	NR	NR	NR	NR	NR	NR
Corsetti et al^25^	7224	20 (4)	2.8 (0.6)	NR	32 (4)	NR	Biopsy (actual)	NR	NR	427	NR
Berg et al^12^	7473	59 (18)	7.9 (2.4)	6714	32 (2)	4.8 (0.3)	Test + (3–5)	759	102	836	125
							Biopsy (actual)	162	22	449	67
Wang et al^26^	NR	176	NR	NR	56	NR	NR	NR	NR	NR	NR
Korpraphong et al^29^	14,483	86	5.9	NR	19	NR	NR	NR	NR	NR	NR
Brem et al^15^	15,318	82 (31)	5.4 (2.0)	13,017	30 (2)	2.3 (0.2)	Test + (0)	2301	150	2063	158
							Biopsy (rec)	610	40	569	44
							Biopsy (actual)	586	38	552	42
Wilczek et al^35^	1668	7	4.2	1645	4	2.4	Test + (SE 3–5)	23	14	23	14
							Biopsy (actual)	11^h^	7	12	7
**Mammography-negative women**											
Kaplan^40^	NR	NR	NR	1862	5 (1)	2.7 (0.5)	Test + (def)^b^	NR	NR	250	134
							Biopsy (rec)	NR	NR	56	30
Crystal et al^38^	NR	NR	NR	1517	7 (0)	4.6 (0)	Test + (def)^c^	NR	NR	90	59
							Biopsy (actual)	NR	NR	38	25
Weinstein et al^46^	NR	NR	NR	363^a^	3 (0)	8.3 (0)	NR	NR	NR	NR	NR
Youk et al^41^	NR	NR	NR	446	11	24.7	Test + (3–5)	NR	NR	134	300
							Test + (4–5)	NR	NR	51	114
Hooley et al^8^	NR	NR	NR	648	3 (1)	4.6 (1.5)	Test + (3–5)	NR	NR	153	236
							Test + (4–5)	NR	NR	38	59
							Biopsy (rec)	NR	NR	64	99
							Biopsy (actual)	NR	NR	63	97
Leong et al^42^	NR	NR	NR	141	2 (1)	14.2 (7.1)	Test + (U3–4)	NR	NR	24	170
							Test + (U4)	NR	NR	14	99
							Biopsy (rec)	NR	NR	14	99
Weigert and Steenbergen^44^	NR	NR	NR	8647	27 (4)	3.1 (0.5)	Test + (3–5)	NR	NR	1196	138
							Test + (4–5)	NR	NR	429	50
							Biopsy (rec)	NR	NR	429	50
Girardi et al^39^	NR	NR	NR	9960	22	2.2	NR	NR	NR	NR	NR
Chang et al^37^	NR	NR	NR	990	5 (2)	5.1 (2.0)	Test + (3–5)	NR	NR	366	370
							Test + (4–5)	NR	NR	84	85
Hwang et al^43^	NR	NR	NR	1349	8 (1)	5.9 (0.7)	NR	NR	NR	NR	NR
Weigert and Steenbergen^45^	NR	NR	NR	10,282	23 (9)	2.2 (0.9)	Test + (3–5)	NR	NR	1310	127
							Test + (4–5)	NR	NR	435	42
							Biopsy (rec)	NR	NR	435	42
Kim et al^48^	NR	NR	NR	3171	9 (2)	2.8 (0.6)	Test + (3–5/md)	NR	NR	831	262
							Test + (4–5/md)	NR	NR	131	41
							Biopsy (rec)	NR	NR	131	41
							Biopsy (actual)	NR	NR	147	46
Tagliafico et al^36^	NR	NR	NR	3231	23 (1)	7.1 (0.3)	Test + (3–5)	NR	NR	145	45
							Test + (4–5)	NR	NR	88	27
							Biopsy (actual)	NR	NR	47	15
Destounis et al^50^	NR	NR	NR	5434	18 (0)	3.3 (0)	Test + (3–5)	NR	NR	194	36
							Test + (4–5)	NR	NR	100	18
							Biopsy (actual)	NR	NR	104	19
Klevos et al^49^	NR	NR	NR	394	0 (0)	0 (0)	Test + (3–5)	NR	NR	69	175
							Test + (4–5)	NR	NR	19	48
							Biopsy (rec)	NR	NR	24	61
							Biopsy (actual)	NR	NR	26	66
Weigert^47^	NR	NR	NR	7459	21 (1)	2.8 (0.1)	Test + (3–5)	NR	NR	727	97
							Test + (4–5)	NR	NR	201	27
							Biopsy (rec)	NR	NR	201	27

*DCIS* ductal carcinoma in situ, *md* modified BI–RADS categorisation (complicated cysts ≤5 mm observed as circumscribed, homogenous and hypoechoic lesions or circumscribed oval-shaped solid masses ≤5 mm without any suspicious US features were downgraded to BI–RADS 2), *MX-* mammography negative women, *NR* not reported, *rec* recommended

^a^ Estimated from proportions

^b^ Defined as: dominant cystic mass, solid mass, areas of architectural distortion or acoustic shadowing

^c^ Defined as: complex cysts or solid lesions

^e^ In most studies, screening test outcomes were recorded using the BI–RADS system

^f^ With or without additional physical examination/clinical breast examination

^g^ Might include some women with abnormal mammography, in parts of MX- breasts. Data were not reported separately for women with MX-

^h^ Eight out of 23 women with abnormal mammography were not referred for assessment after an ultrasound examination, and were counted in the “healthy” group.

**Fig. 3:**
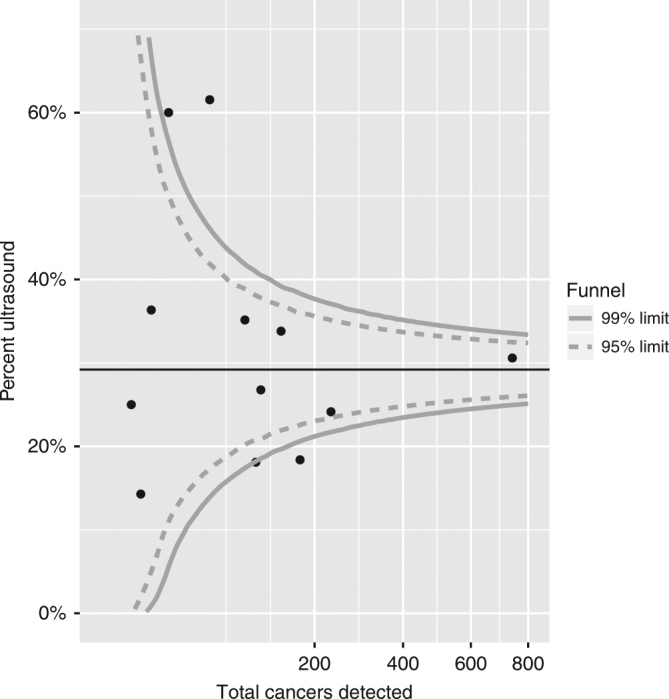
Funnel plot of the percentage of cancers detected by ultrasound against the total number of cancers detected (based on 12 studies reporting detection by both screening modalities)

A funnel plot is shown in Fig. 3. There is a small suggestion of publication bias due to the two small studies reporting large effects, but this was tempered a little by a couple of larger studies with smaller effect sizes.

Subgroups were investigated to assess whether the variation between studies could be explained by (1) studies that included BI–RADS density 2 as ‘dense’; (2) study year (a proxy for digital vs. film mammography); or (3) extent of other risk factors. Although small differences were observed, these did not appear to explain the variation between studies (Fig. 2b-d).

**Fig. 4:**
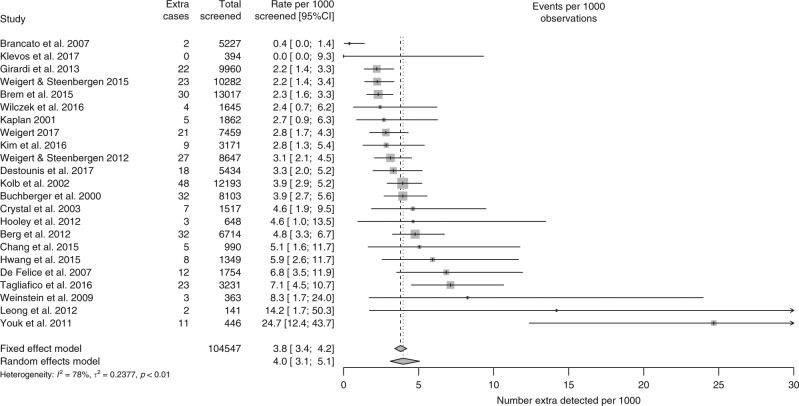
Extra detection of cases of breast cancer per 1000 women with negative mammography (based on 23 studies reporting detection in mammographically negative women). CI confidence interval

Per 1000 screens in women with negative mammography, ultrasound detected on average ca. 4 additional cases of breast cancer (FE: 3.8, 95% CI: 3.4–4.2; RE: 4.0, 95% CI: 3.1–5.1; as shown in Fig. 4, this estimate was based on all 23 studies that reported numbers of screened women with negative mammography). This was slightly higher, 5 per 1000, in two studies of women with additional risk factors.^12,46^ In the 17 studies separating DCIS from invasive cases, approximately 13% (32/248) were DCIS.

Although for all studies with reported data a large number of cases were detected by both screening methods (Supplementary Table 2), there were a considerable number of cases that were detected by only one method.

Where data were available, there appeared to be no strong and significant correlation between the number of cancers detected by mammography and those additionally detected only by ultrasound, neither when additional detection by ultrasound was considered in absolute (*ρ* weighted by number of women in the study = 0.23, *P* = 0.62) nor in relative (weighted *ρ* = –0.48, *P* = 0.27) terms.

### Impact on recall for assessment

Recall for assessment after supplemental ultrasound screening could be compared to recall after mammography on data from three studies,^12,15,35^ two from the USA and one from Sweden. Here, supplemental ultrasound approximately doubled the number of screens with non-normal findings (Table 2). In two studies, the number of biopsies was also doubled,^12,35^ whereas in another study,^15^ it was almost trebled. Both studies from the USA had an already high mammography abnormality rate, 10% (BI–RADS 3 to 5)^12^ and 15% (BI–RADS 0, roughly equivalent to BI–RADS 4–5 in other studies).^15^ Interestingly, mammographic abnormalities were much more infrequent in the Swedish study, just above 1%,^35^ which was also lower than the data reported for the routine screening programme (~3%).^51^ Another study from the USA also reported a sharp, 76%, increase in the number of biopsies.^27^

Per 1000 mammography negative screens, ultrasound was positive in 110–130 screens (FE: 131, 95% CI: 128–134; RE: 109, 95% CI: 80–145) when a positive screen was defined as BI–RADS categories 3–5. Ultrasound would typically prompt a recall for assessment, defined as BI–RADS categories 4–5, in on average 85 screens per 1000 (FE: 95% CI: 83–88), although this was lower in smaller studies as evidenced by the RE estimate, 45 per 1000 (95% CI: 26–75). Approximately 50 per 1000 mammography negative screens were followed by a recommendation for a biopsy (FE: 47/RE: 53). Almost all of the women concerned actually had one, though that was less frequently the case in the smaller studies (FE: 40/FE: 28). All these proportions varied considerably among studies. Of the 13 studies with reported data, only one included women with additional risk factors, so the higher-than-average risk cannot explain the high proportions of women with non-normal ultrasound findings.

The data did not suggest a relationship between an (increased) number of women referred for assessment and an (increased) cancer detection. The correlation between the proportion of screens with non-normal ultrasound findings (BI–RADS 3–5 or equivalent) and the extra detected number of cancer cases per 1000 mammography negative screens was weak (*ρ* weighted by number of women with negative mammography = 0.25, *P* = 0.32). The correlations with the proportions of screens with more severely abnormal ultrasound findings (BI–RADS 4–5 or equivalent), and of screens followed by a biopsy, were also not significant (weighted *ρ* = 0.03 and *P* = 0.93, and *ρ* = 0.35 and *P* = 0.17, respectively).

### Quality of the studies and of their reporting

An evaluation of the quality of the studies and of their reporting using the QUADAS–2 framework revealed some potential issues with universal applicability of the findings and a potential for bias in terms of patient selection and (the interpretation of) the index tests (Supplementary table 3). These were related to e.g. an inclusion of women with scattered fibroglandular tissue among those with “dense” breasts, (retrospective) interpretation of screening tests with knowledge from other imaging methods, and exclusion of mammography negative but palpable tumours after an adjunct clinical breast examination, as this is not a standard screening procedure in settings such as the UK.

### Time investment for ultrasound examinations

The reporting of time spent performing a screening ultrasound differed by study, so no meta-analysis was undertaken for this outcome. The time needed for an ultrasound appeared to be around 10 min per woman on average, although the estimates were highly variable and ranged from mean/median of 5–20 (Supplementary Table 4). Additionally, Hooley and colleagues^8^ reported that the (routine) ultrasound appointments were scheduled at 45-min intervals.

## Discussion

### Main findings

In its latest review from 2016, International Agency for Research on Cancer (IARC) concluded that there is limited evidence for an increased detection of breast cancer using supplemental ultrasound in women with dense breasts, citing the lack of randomised controlled trials and study design heterogeneity among the reasons.^4^ Our meta-analysis, focusing on the most recent studies, showed an on average 40% increase in the detection of asymptomatic breast cancers. Cases missed by mammography were detected by ultrasound in all but one (underpowered) study.

There are still no data on whether the additional detection by ultrasound improves mortality from breast cancer, which is in line with the conclusions made by IARC’s review.^4^ The cases detected only by ultrasound were frequently relatively small, however, the majority were invasive cancers. Some studies reported interval cancers, but for the time being those data appear less informative, as the length of follow-up and the completeness of the ascertainment differed substantially between studies. In the future, it would be helpful to see results from a large cohort with complete ascertainment of interval cancer cases, as this would give us an estimate of the effect of supplementary ultrasound on screening programme sensitivity and its ability to reduce breast cancer mortality.

### Clinical implications

Even though in women with dense breasts ultrasound detects cancers that are missed by mammography (Supplementary Table 2), ultrasound should be considered as a supplemental rather than a stand-alone screening method. In studies where all women underwent both screening tests, roughly 10–30% of all screen-detected cases were detectable only on mammography.

Currently in England, women 50–70 years of age with dense breasts are screened with digital mammography every three years, same as women with fatty breasts. In 2015–2016, 1.8 million women were screened, with on average 410 women referred for assessment and 82 having a breast cancer detected per every 10,000.^52^ This means that about 5 women were referred per diagnosed cancer case. These statistics are unfortunately not reported by breast density but the prevalence of at least heterogeneously dense breasts (BI–RADS 3–4) among screened women appears to be about 40%.^53–58^ Women with dense breasts have roughly twice the risk of breast cancer than those with fatty breasts,^59^ and we assume that the risk of an abnormal mammogram is similarly increased. With these estimates in mind, it can be approximated that among every 10,000 screened women 4000 have dense breasts (Supplementary Table 5A). Of the 410 referred in total, 234 referrals would be in those with dense breasts, as would be 47 among the 82 cancers detected with mammography. Based on our meta-analysis, 3766 ultrasound examinations in mammography negative women would lead to a detection of an additional 15–19 cancer cases, of which 2–3 would be DCIS. The absolute number of additional cancer cases will depend on the underlying risk in the population but both relative and absolute meta-analysis results give similar numbers for this example. This would necessitate an additional 234 referrals (+100%), or 13–16 per additionally detected cancer case. Mammography screening of women aged 50–70 years with supplemental ultrasound for 40% with dense breasts would, therefore, necessitate 10,000 mammograms, 3766 ultrasound examinations (lasting, on average, around 10 min) and 644 referrals for assessment (57% more than with mammography alone), and would detect 97–101 breast cancers (18–23% more than mammography alone).

These calculations suggest that there are important capacity considerations for an introduction of ultrasound as a supplementary screening method for women with dense breasts. At present, ultrasound is used as part of assessment after positive mammography, i.e. in 410/10,000 screened women. Hence, the use of ultrasound in screening for 40% of the target population would require a ten-fold increase in the ultrasound availability. To lessen the impact on service providers, supplemental ultrasound screening could instead be considered for smaller subgroups of women with a particularly increased risk of breast cancer. As an example, ultrasound screening could be reserved for the approximately 10% women with extremely dense breasts whose relative risk of breast cancer is increased approximately threefold compared to the rest of the population.^53,59,60^ Assuming the same supplemental detection with ultrasound as in the meta analysis, this strategy would require 898 instead of 3766 ultrasound examinations per 10,000 screened women and a 25% overall increase in referrals for assessment (an additional 103/10,000). A conservative estimate of the expected increase in the detection rate, based on the average effect found in our meta-analysis, would be in the order of 10% (an additional 8/10,000; Supplementary Table 5B).

From the above calculations, it is evident that although the addition of ultrasound would increase the number of assessments, the major call on resources would be the performance of the ultrasound examinations. This could potentially be kept to a manageable level by use of a high density threshold.

At present, 31 women per 10,000 screened have an interval breast cancer diagnosed after negative mammography.^61^ It is likely that supplementary ultrasound could help decrease this risk by detecting cancers already at screening, but it is not yet clear by how much because the extent of overdiagnosis for now remains uncertain. Assuming that, like in mammography,^62^ also here overdiagnosis can explain only a small proportion of cases, the additional detection by supplementary ultrasound (estimated above at 19 or 8 per 10,000), could prove to be clinically meaningful in decreasing the overall interval cancer rate.

### Strengths and weaknesses

We did a thorough systematic search in the leading medical database, and, additionally, hand-searched all identified reviews and similar secondary literature. We used pre-specified selection criteria and excluded studies that did not describe routine screening settings.

Although none of the studies was a randomised trial, both mammography and ultrasound testing in the selected studies were undertaken sequentially in the same women. This means that all women acted as their own controls, thereby accounting for between-patient variability. However, we cannot exclude a potential study effect on mammography interpretation particularly in the more complex cases, originating from the radiologists being aware that ultrasound will form part of the screening evaluation.

Nevertheless, several studies identified in our search had to be excluded from the review as they did not report the data separately for asymptomatic women undergoing screening, or by their breast density. This suggests that a substantial amount of the relevant data may have remained unreported, however, funnel plot analysis suggested only a small effect of a publication bias.

Although all studies described asymptomatic women, there were important differences in their study designs. First, while most studies defined dense breasts as those with at least heterogeneous density (≥50% fibroglandular tissue), a handful of studies reported data for women with at least scattered density (25–49% fibroglandular tissue) where the risk of breast cancer is generally lower^59^ and mammography tends to be more sensitive.^63^ The inclusion of women with scattered density appears to have slightly diluted the beneficial effect of the ultrasound (proportion of cancers detected by ultrasound 0.23 with BI–RADS 2-4 vs. 0.31 with BI–RADS 3–4). Second, intensive additional imaging, including not just ultrasound but also e.g. magnetic resonance, may be a sensible option for women with additional risk factors such as those who are BRCA 1/2 gene carriers or have a high estimated lifetime risk of breast cancer.^64^ The inclusion of studies focusing only on women with additional risk factors did not seem to substantially alter our results (proportion of cancers detected by ultrasound with additional risk factors 0.29 vs. 0.33 when women were not selected based on additional risk factors). It should be noted, however, that at least a small proportion of high-risk women were included in virtually all studies. Third, older studies used film-screen mammography, which was shown in some studies to be less sensitive in dense breasts than digital mammography.^55^ Adding ultrasound appeared to be slightly more beneficial after film-screen mammography (proportion of cancers detected by ultrasound 0.31 vs. 0.25 in digital mammography). Multiple studies are now underway to further improve the detection of breast cancer with mammography. An example of new mammography-based technologies is supplemental tomosynthesis, which appears to significantly improve the overall detection of breast cancer,^65^ including that among women with dense breasts.^66^ Only two studies used the newer automated ultrasound technology;^15,35^ in both studies, the extra detection was close to the pooled estimate. Ultrasound screening was undertaken by radiographers in one large study using hand-held devices.^26^ The additional detection of breast cancer was slightly lower in this study than in the pooled estimate, suggesting that, for radiologist-operated and radiologist-read hand-held ultrasound screening, the pooled estimate is on the conservative side. Another factor that may have affected the comparisons between the studies is the proportion of studied women undergoing prevalence (first) and incidence (any subsequent) screening rounds. In mammography screening, breast cancer detection and the frequency of referral for assessment are both lower in the incidence rounds.^52^ The same trend was suggested for ultrasound screening in the study by Berg and colleagues,^12^ where ultrasound detected 70% more cases than mammography in the prevalence round (14 only by ultrasound vs. 20 by mammography), and thereafter 56% (9 vs. 16) and 39% (9 vs. 23) more cases in the first and the second incidence rounds, respectively. The frequency of positive ultrasound examinations without an underlying cancer about halved from the prevalence to the incidence rounds, with 16% and 8%, respectively. A more detailed reporting of the outcomes by screening round from other studies would be informative. Additionally, BI-RADS density classification has changed over time, with the latest edition published in 2013 effectively lowering the threshold for classifying breasts as dense.^67^ Finally, the studies differed in the degree to which ultrasound image interpretation could be influenced by knowledge of mammography imaging and vice versa (although this detail was not consistently reported), and in how they selected their study populations, e.g. by whether screening was organised or opportunistic. All these factors may have led to slightly different selections of women in terms of their risk profile, and this heterogeneity needs to be taken into account in the interpretation of the results.

## Conclusion

Studies have consistently shown an increased detection by supplementary ultrasound of predominantly small but invasive breast cancers in women with dense breasts. The feasibility of this screening method in routine practice might be at present limited given its resource use, although the strain on the health care capacities might be manageable by a careful targeting of the highest-risk women among those with dense breasts.

## Electronic supplementary material

Supplementary Tables

## Availability of data and material

The study was based entirely on previously published data.
