# Supplementary material for: Addition of ultrasound to mammography in the case of dense breast tissue: systematic review and meta-analysis
Source: Br J Cancer. 2018 May 8;118(12):1559–70. doi: 10.1038/s41416-018-0080-3 (PMC6008336; doi:10.1038/s41416-018-0080-3)
Supplement: Supplementary file 1 — Supplementary Tables [file 41416_2018_80_MOESM1_ESM.docx]

**SUPPLEMENTARY MATERIAL**

Supplementary table 1. Description of the processes in the studies.

| **Authors, publication year** | **CBE** | **Blinding of screening diagnosis** |  | **Definition of positive MX (BI–RADS)** | **Definition of positive US (BI–RADS)^a^** | **MX and US on same day** |
| --- | --- | --- | --- | --- | --- | --- |
|  |  | **MX to US** | **US to MX** |  |  |  |
| Buchberger et al. 2000 |  |  |  | NR | NR | NR |
| Kuhl et al. 2000 | Yes |  | Yes | 3-5 | 0 or 3-5 | NR |
| Kaplan 2001 | Yes |  |  | NR | 3-5, but also^u^ | No (in “most”) |
| Kolb et al. 2002 | Yes |  |  | NR | NR | NR |
| Crystal et al. 2003 | Yes |  |  | NR | Suspicious for malignancy or complex cyst^u^ | Mostly yes |
| Brancato et al. 2007 | Yes |  |  | NR | U3-5 | In ≤1 month |
| De Felice et al. 2007 |  |  |  | 4-5 | NR | Yes |
| Sardanelli et al. 2007 | Yes |  | Yes | 3-5 | 3-5 | In ≤2 months |
| Weinstein et al. 2009 | Yes |  |  | 0 and 4-5 if unresolved or no negative biopsy | 0, 3-5 | Within 180 days |
| Bae et al. 2011 | Yes |  |  | 3-5 | 3-5 | NR |
| Corsetti et al. 2011 |  |  |  | NR | 3-5 | NR |
| Youk et al. 2011 | Yes |  |  | 3-5 | 3-5 | NR |
| Berg et al. 2012 |  |  | Yes | 3-5^f^ | 3-5^f^ | In ≤2 weeks |
| Hooley et al. 2012 | Yes |  |  | 3-5 | 3-5, but also^u^ | No |
| Leong et al. 2012 | Yes |  |  | 3-5 | U3-U4 | NR |
| Weigert & Steenbergen 2012 |  |  |  | 3-5 | 3-5 | No |
| Girardi et al. 2013 | Yes | No |  | NR^v^ | 0, 3-5, but also^u^ | Yes |
| Wang et al. 2013 |  |  |  | 4-5 | 4-5 | NR |
| Korpraphong et al. 2014 |  | No |  | 4-5 | 4-5 | Yes |
| Brem et al. 2015 |  |  |  | 0 | 0 | Yes |
| Chang et al. 2015 |  | No |  | 3-5 | 3-5 | Yes |
| Hwang et al. 2015 |  |  |  | 3-5 | 3-5 | Yes |

Supplementary table 1 (continued). Description of the processes in the studies.

| **Authors, publication year** | **CBE** | **Blinding of screening diagnosis** |  | **Definition of positive MX (BI–RADS)** | **Definition of positive US (BI–RADS)^a^** | **MX and US on same day** |
| --- | --- | --- | --- | --- | --- | --- |
|  |  | **MX to US** | **US to MX** |  |  |  |
| Weigert & Steenbergen 2015 |  |  |  | 3-5 | 3-5 | No |
| Kim et al. 2016 |  |  |  | 3-5 | 3-5 | In ≤3 months |
| Tagliafico et al. 2016 |  |  |  | NR | 3-5 | Yes |
| Wilczek et al. 2016 |  |  |  | SE code 3-5 | SE code 3-5 | Yes |
| Destounis et al. 2017 |  |  |  | 3-5 | 3-5 | In on avg. 24 days |
| Klevos et al. 2017 |  |  |  | 3-5 | 3-5 | In ≤6 months |
| Weigert 2017 |  |  |  | 3-5 | 3-5 | No |

Abbreviations: BI-RADS grade 3 abnormality: probably benign, typically requiring a follow-up screen but not an immediate referral for assessment. BI-RADS grade 4 or 5 abnormality: at least highly suspicious for abnormality, typically requiring an immediate referral for assessment. CBE=clinical breast examination. MX=mammography. NR=not reported. US=ultrasound.

^a^ Includes women referred for assessment and, if reported, women referred for short-term follow-up.

^f^ With recommendation for other than routine screening.

^u^ Based on criteria by Stavros and colleagues and reviewed by Baker and Soo. US-detected mass had ≥2 of the following signs: spiculation, angular margins, marked hypoechogenecity, shadowing, duct extension, branch pattern, microlobulation. Included women with 6-month follow-up.

^v^ When suspicious lesions were detected on US alone, original MX slides were reviewed to determine whether the lesion was retrospectively visible. Cancer cases with minimal suspicions on MX were attributed to MX.

Supplementary table 2. Reported data on overlapping and non-overlapping cases where a breast cancer was detected by mammography, ultrasound, or both.

Kuhl et al. 2000

|  | Ultrasound |  |  |
| --- | --- | --- | --- |
| Mammography | Positive | Negative | Total |
| Positive | 2 | 1 | 3 |
| Negative | 1 |  |  |
| Total | 3 |  |  |

Sardanelli et al. 2007

|  | Ultrasound |  |  |
| --- | --- | --- | --- |
| Mammography | Positive | Negative | Total |
| Positive | 6 | 0 | 6 |
| Negative | 1 |  |  |
| Total | 7 |  |  |

Kolb et al. 2002

|  | Ultrasound |  |  |
| --- | --- | --- | --- |
| Mammography | Positive | Negative | Total |
| Positive | 62 | 32 | 94 |
| Negative | 48 |  |  |
| Total | 110 |  |  |

Brem et al. 2015

|  | Ultrasound |  |  |
| --- | --- | --- | --- |
| Mammography | Positive | Negative | Total |
| Positive | 65 | 17 | 82 |
| Negative | 30 |  |  |
| Total | 95 |  |  |

Berg et al. 2012

|  | Ultrasound |  |  |
| --- | --- | --- | --- |
| Mammography | Positive | Negative | Total |
| Positive | 59 | 0 | 59 |
| Negative | 32 |  |  |
| Total | 91 |  |  |

Wang et al. 2013

|  | Ultrasound |  |  |
| --- | --- | --- | --- |
| Mammography | Positive | Negative | Total |
| Positive | 155 | 21 | 176 |
| Negative | 56 |  |  |
| Total | 211 |  |  |

Korpraphong et al. 2014

|  | Ultrasound |  |  |
| --- | --- | --- | --- |
| Mammography | Positive | Negative | Total |
| Positive | 55 | 31 | 86 |
| Negative | 19 |  |  |
| Total | 74 |  |  |

Supplementary table 3. Evaluation of study quality following the QUADAS-2 framework.

| **Study and publication year** | **Risk of bias** | | | | **Applicability concerns** | | | |
| --- | --- | --- | --- | --- | --- | --- | --- | --- |
|  | **Patient selection** | **Index test(s)** | **Reference standard** | **Flow and timing** | | **Patient selection** | **Index test(s)** | **Reference standard** |
| Buchberger et al. 2000 | - | + | + | + | | - | - | + |
| Kuhl et al. 2000 | ? | + | + | ? | | + | + | + |
| Kaplan 2001 | ? | + | + | + | | ? | - | + |
| Kolb et al. 2002 | - | + | + | + | | - | + | + |
| Crystal et al. 2003 | - | - | + | + | | - | - | + |
| Brancato et al. 2007 | - | + | + | - | | ? | ? | + |
| De Felice et al. 2007 | + | + | + | + | | + | + | + |
| Sardaneli et al. 2007 | ? | + | + | + | | + | + | + |
| Weinstein et al. 2009 | ? | + | + | + | | + | - | ? |
| Bae et al. 2011 | + | ? | + | + | | + | - | + |
| Corsetti et al. 2011 | + | + | + | + | | + | + | + |
| Youk et al. 2011 | - | + | + | - | | - | - | + |
| Berg et al. 2012 | ? | + | + | + | | + | + | + |
| Hooley et al. 2012 | ? | + | + | + | | ? | - | + |
| Leong et al. 2012 | ? | + | + | + | | + | + | + |
| Weigert & Steenbergen 2012 | ? | + | + | ? | | ? | + | + |
| Girardi et al. 2013 | + | - | + | + | | - | - | + |
| Wang et al. 2013 | + | + | + | ? | | ? | + | - |
| Korpraphong et al. 2014 | + | + | + | + | | - | + | + |
| Brem et al. 2015 | + | + | + | + | | + | + | + |
| Chang et al. 2015 | - | + | + | + | | - | + | + |
| Hwang et al. 2015 | - | + | + | + | | ? | + | + |
| Weigert & Steenbergen 2015 | ? | + | + | ? | | ? | + | + |
| Kim et al. 2016 | ? | + | + | ? | | ? | - | + |
| Tagliafico et al. 2016 | + | + | + | + | | - | + | + |
| Wilczek et al. 2016 | + | + | + | ? | | + | + | + |
| Destounis et al. 2017 | ? | + | + | ? | | ? | + | + |
| Klevos et al. 2017 | ? | + | + | ? | | ? | + | + |
| Weigert 2017 | ? | + | + | ? | | ? | + | + |

Abbreviations: +=low risk. -=high risk. ?=unclear.

Supplementary table 4. Duration of ultrasound examinations as reported in the original publications.

| **Study** | **Reported mean or median duration of ultrasound examination** |
| --- | --- |
| Buchberger et al. 2000 | 10-15 minutes |
| Kaplan 2001 | 10 minutes (range: 7-20) |
| Kolb et al. 2002 | Mean: 4 minutes (range: 2-11) |
| Crystal et al. 2003 | 7 minutes (range: 4-15) |
| Berg et al. 2012 | 1-17 minutes (range: 1-166) |
| Hooley et al. 2012 | Examination appointments scheduled at 45-minute intervals |
| Leong et al. 2012 | 13 (±6) minutes for bilateral and 11 (±1) minutes for unilateral US |
| Weigert and Steenbergen 2012 | 10-20 minutes |
| Girardi et al. 2013 | 7 minutes (range: 4-25) |
| Brem et al. 2015 | 15 minutes |
| Chang et al. 2015 | 15-20 minutes |
| Hwang et al. 2015 | 10 minutes (range: 5-20) |
| Wilczek et al. 2016 | 15 minutes for the examination, 5-7 minutes for reading |

Supplementary table 5. Estimated effect of using ultrasound for mammography negative women with dense breasts in England. Per 10,000 women screened in a general population.

Table S5A. Ultrasound is offered to women with heterogeneously or extremely dense breasts and negative mammography.

|  | **Number** | **Notes** |
| --- | --- | --- |
| Screened with mammography, general population | 10,000 | Of these women, 4.1% have abnormal mammography and 8.2/1000 have a screen-detected breast cancer^52^ |
| **Women with heterogeneously or extremely dense breasts** |  |  |
| N | 4000 | Approximate average in the literature: 40%^53-58^ |
| With positive mammography (=number of referrals for assessment) | 234 | Estimated proportion of abnormal mammography in women with dense breasts: 5.9%, calculated based on the following assumptions:   - Proportion with abnormal mammography in the general population: 4.1%^52^ - Prevalence of dense breasts in the screened population: 40% - Assume mammographic abnormalities are twice as frequent in dense breasts compared to fatty breasts; this assumption is based on the literature concerning the risk of cancer (see below) |
| Cancers detected by mammography | 47 | Estimated cancer detection rate in women with dense breasts: 11.8/1000, calculated based on the following assumptions:   - Detection of breast cancer at screening in the general population aged 50-70 years: 8.2/1000^52^ - Prevalence of dense breasts in the screened population: 40% - Assume breast cancer is detected twice as frequently in dense breasts compared to fatty breasts^54,56,58,59^ |
| With negative mammography (= number of ultrasound examinations) | 3766 | See above |
| Extra cancers detected by ultrasound (calculation method A) | 15 | Additional detection of 4/1000 in mammography negative women (from meta-analysis) |

Table S5A (continued). Ultrasound is offered to women with heterogeneously or extremely dense breasts and negative mammography.

|  | **Number** | **Notes** |
| --- | --- | --- |
| Extra cancers detected by ultrasound (calculation method B) | 19 | Additional detection of ca. 40% more than mammography (from meta-analysis) |
| Extra DCIS detected by ultrasound | 2-3 | 0.6 of the additional 4 breast cancers detected by ultrasound are DCIS (from meta-analysis) |
| Extra referrals for assessment by ultrasound | 234 | Approximately twice as many referrals with mammography combined with supplementary ultrasound screening compared to mammography alone (from meta-analysis) |
| Total for mammography alone, per 10,000 women | 10,000 mammographies  0 ultrasounds  410 referrals  82 detected cancers |  |
| Total for mammography combined with supplementary ultrasound for women with dense breasts and negative mammography, per 10,000 women | 10,000 mammographies  3766 ultrasounds  644 referrals  97-101 detected cancers | Consequence:  = no change  = needed extra capacity  = 57% more than mammography alone  =18-23% more than mammography alone |

Table S5B. Ultrasound is offered to women with extremely dense breasts and negative mammography.

|  | **Number** | **Notes** |
| --- | --- | --- |
| Screened with mammography, general population | 10,000 | Of these women, 4.1% have abnormal mammography and 8.2/1000 have a screen-detected breast cancer^52^ |
| **Women with extremely dense breasts** |  |  |
| N | 1000 | Literature estimates: approximately 5-10%^53,54,60^ |
| With positive mammography (=number of referrals for assessment) | 103 | Estimated proportion of abnormal mammography in women with extremely dense breasts: 10.3%, calculated based on the following assumptions:   - Proportion with abnormal mammography in the general population: 4.1%^52^ - Prevalence of extremely dense breasts in the screened population: 10% - Assume mammographic abnormalities are three times as frequent in extremely dense breasts compared to heterogeneously or less dense breasts; this assumption is based on the literature concerning the risk of cancer (see below) |
| Cancers detected by mammography | 21 | Estimated cancer detection rate in women with extremely dense breasts: 20.5/1000, calculated based on the following assumptions:   - Detection of breast cancer at screening in the general population aged 50-70 years: 8.2/1000^52^ - Prevalence of extremely dense breasts in the screened population: 10% - Assume breast cancer is detected three times as frequently in extremely dense breasts compared to heterogeneously or less dense breasts^54,56,58,59^ |
| With negative mammography (= number of ultrasound examinations) | 898 | See above |
| Extra cancers detected by ultrasound (calculation method A) | 4 | Additional detection of 4/1000 in mammography negative women (from meta-analysis; this is a conservative estimate for women with extremely dense breasts) |
| Extra cancers detected by ultrasound (calculation method B) | 8 | Additional detection of ca. 40% more than mammography (from meta-analysis; this is a conservative estimate for women with extremely dense breasts) |

Table S5B (continued). Ultrasound is offered to women with extremely dense breasts and negative mammography.

|  | **Number** | **Notes** |
| --- | --- | --- |
| Extra referrals for assessment by ultrasound | 103 | Approximately twice as many referrals with mammography combined with supplementary ultrasound screening compared to mammography alone (from meta-analysis) |
| Total for mammography alone, per 10,000 women | 10,000 mammographies  0 ultrasounds  410 referrals  82 detected cancers |  |
| Total for mammography combined with supplementary ultrasound for women with extremely dense breasts and negative mammography, per 10,000 women | 10,000 mammographies  898 ultrasounds  513 referrals  86-90 detected cancers | Consequence:  = no change  = needed extra capacity  = 25% more than mammography alone  = 5-10% more than mammography alone |
